# DNA Damage Response Is Involved in the Developmental Toxicity of Mebendazole in Zebrafish Retina

**DOI:** 10.3389/fphar.2016.00057

**Published:** 2016-03-14

**Authors:** Shota Sasagawa, Yuhei Nishimura, Tetsuo Kon, Yukiko Yamanaka, Soichiro Murakami, Yoshifumi Ashikawa, Mizuki Yuge, Shiko Okabe, Koki Kawaguchi, Reiko Kawase, Toshio Tanaka

**Affiliations:** ^1^Department of Molecular and Cellular Pharmacology, Pharmacogenomics and Pharmacoinformatics, Mie University Graduate School of MedicineTsu, Japan; ^2^Mie University Medical Zebrafish Research CenterTsu, Japan; ^3^Department of Systems Pharmacology, Mie University Graduate School of MedicineTsu, Japan; ^4^Department of Omics Medicine, Mie University Industrial Technology Innovation InstituteTsu, Japan; ^5^Department of Bioinformatics, Mie University Life Science Research CenterTsu, Japan

**Keywords:** systems toxicology, benzimidazole, ATM, DNA damage response, developmental toxicity, zebrafish

## Abstract

Intestinal helminths cause iron-deficiency anemia in pregnant women, associated with premature delivery, low birth weight, maternal ill health, and maternal death. Although benzimidazole compounds such as mebendazole (MBZ) are highly efficacious against helminths, there are limited data on its use during pregnancy. In this study, we performed *in vivo* imaging of the retinas of zebrafish larvae exposed to MBZ, and found that exposure to MBZ during 2 and 3 days post-fertilization caused malformation of the retinal layers. To identify the molecular mechanism underlying the developmental toxicity of MBZ, we performed transcriptome analysis of zebrafish eyes. The analysis revealed that the DNA damage response was involved in the developmental toxicity of MBZ. We were also able to demonstrate that inhibition of ATM significantly attenuated the apoptosis induced by MBZ in the zebrafish retina. These results suggest that MBZ causes developmental toxicity in the zebrafish retina at least partly by activating the DNA damage response, including ATM signaling, providing a potential adverse outcome pathway in the developmental toxicity of MBZ in mammals.

## Introduction

Helminth infections are highly prevalent and affect 44 million pregnancies, globally, each year (reviewed in Mpairwe et al., 2014; Salam et al., 2015). Intestinal helminths feed on host blood and cause iron-deficiency anemia. Anemia during pregnancy is associated with risk of dying during pregnancy and delivering low birth weight babies. Benzimidazole compounds such as mebendazole (MBZ) and albendazole (ABZ) are highly efficacious anthelminthic; however, the data about their use in pregnancy are limited.

Many benzimidazole compounds, including MBZ and ABZ, have been shown to be teratogenic in animals (reviewed in Dayan, 2003). The US Food and Drug Administration (FDA) has classified MBZ and ABZ in Pregnancy category C (Law et al., 2010). MBZ and ABZ bind to the colchicine-binding domain of tubulin, resulting in the inhibition of tubulin polymerization (Morgan et al., 1993; Xu et al., 2002). Although benzimidazole compounds have high affinities to tubulin in helminths, they also bind to tubulin in mammalian cells. Therefore, inhibition of tubulin polymerization has been suggested as the primary mechanism in developmental toxicity of MBZ and ABZ (Whittaker and Faustman, 1992); however, the exact mechanism underlying the developmental toxicity of these benzimidazole compounds remains largely unknown.

Zebrafish is considered as an excellent model organism in various biomedical fields, including developmental toxicology (Sipes et al., 2011; Bal-Price et al., 2012; Behl et al., 2015; Nishimura et al., 2015a, 2016). The development process is highly conserved across vertebrate species, making zebrafish development largely comparable to that of mammals. Zebrafish is also highly amenable to transcriptome analysis and various imaging analyses. Transcriptomic analysis is currently the best-established approach for identifying perturbed biological networks, thereby gaining mechanistic insight into the system's response to an exposure (Sturla et al., 2014). Zebrafish remain transparent from fertilization up to 2 days post-fertilization (dpf). Development of pigmentation can also be suppressed by treating zebrafish larvae with 1-phenyl-2-thyourea, an inhibitor of melanin synthesis. Several pigmentless zebrafish lines are available for laboratory studies. These features allow unobstructed observation of morphological changes of various cells and tissues during the earlier development stages by using fluorescence technology (Watanabe et al., 2012; Nishimura et al., 2013; Sasagawa et al., 2016).

Several studies have been performed to assess developmental toxicity of MBZ and ABZ in zebrafish (Kitambi et al., 2009; Carlsson et al., 2011, 2013). Zebrafish exposed to ABZ from 0 to 6 dpf showed visceral, craniofacial, and bone defects, which are similar to the developmental toxicity of ABZ in rodents (Carlsson et al., 2011, 2013). Zebrafish exposed to MBZ from 0 to 2.5 dpf showed defects of blood vessels in retina and trunk, and the loss of neuronal architecture in the eye (Kitambi et al., 2009). The mechanisms underlying the developmental toxicity of these benzimidazole compounds, however, remain largely unknown.

The current study focused on the findings of Kitambi et al. (2009), which demonstrated that MBZ adversely affected the organization of retinal layers. We hypothesized that some developmental stages may be highly susceptible to the toxicity of MBZ in the zebrafish retina. To examine the critical stage, we performed fluorescent *in vivo* imaging of zebrafish retinal layers using a coumarin derivative (Watanabe et al., 2010). The fluorescent *in vivo* imaging revealed that MBZ causes disorganization of retinal layers in zebrafish during 2 and 3 dpf. To reveal the adverse outcome pathway in the developmental toxicity of MBZ, further transcriptome analysis of zebrafish eyes was performed. The DNA damage response, including the ATM pathway, was shown to be involved in the developmental toxicity of MBZ.

## Materials and methods

### Ethics statement

This study was carried out in strict accordance with Japanese law, including the Humane Treatment and Management of Animals Act (2014), Standards Relating to the Care and Management of Laboratory Animals and Relief of Pain (2013), and the Guidelines for Proper Conduct of Animal Experiments (Science Council of Japan, 2006). All experiments were performed under 2-phenoxyethanol anesthesia, and all efforts were made to minimize suffering.

### Compounds

MBZ, ABZ, and nocodazole (NCZ) were obtained from Sigma-Aldrich (St. Louis, MO, USA). Butyl 2-({2-[(methoxycarbonyl)amino]-1H-benzimidazol-5-yl}carbonyl) benzoate (BBC) was obtained from Namiki Shoji (Tokyo, Japan). ZMA462, formerly called DIBPBC (Watanabe et al., 2010), was obtained from Canon Inc. (Tokyo, Japan). Stock solutions for these compounds were prepared by dissolving in dimethyl sulfoxide (DMSO; Nacalai Tesque, Kyoto, Japan). 2-Phenoxyethanol was obtained from Wako Chemical (Osaka, Japan).

### Zebrafish

Zebrafish AB line was obtained from ZIRC (Eugene, OR, USA) (Varga, 2011) and an albino line (Kelsh et al., 1996) was obtained from the Max Planck Institute for Developmental Biology (Tübingen, Germany). Zebrafish were bred and maintained according to previously described methods (Westerfield, 2007; Nishimura et al., 2016). Briefly, zebrafish were raised at 28.5 ± 0.5°C with a 14/10 h light/dark cycle. Embryos were obtained and cultured in 0.3 × Danieau's solution (19.3 mM NaCl, 0.23 mM KCl, 0.13 mM MgSO_4_, 0.2 mM Ca(NO_3_)_2_, 1.7 mM HEPES, pH 7.2) until 6 dpf.

### *In vivo* imaging of the zebrafish retina

Zebrafish were exposed to benzimidazole compounds at indicated concentrations and for indicated time periods. The tests were performed in 6-well plates with 10 embryos per well. After the exposure to benzimidazole compounds, the vital staining of zebrafish with a fluorescent dye, ZMA462 (Watanabe et al., 2010), was performed to visualize the retinal layers. In the vital staining, the inner plexiform layer (IPL) and outer plexiform layer (OPL) are imaged with strong fluorescence, whereas the ganglion cell layer (GCL), inner nuclear layer (INL), and outer nuclear layer (ONL), appear reticulated. The IPL and OPL are synaptic layers that contain neuronal projections from the INL and GCL, and from the ONL and INL, respectively. The strong fluorescence in the IPL and OPL and reticular staining of the GCL, INL, and ONL suggest that ZMA462 may stain the plasma membranes of neuronal cells in the zebrafish retina (Watanabe et al., 2010).

After bathing the zebrafish in a medium containing 1 μg/mL ZMA462 for 30 min at 28.5°C, zebrafish were washed, anesthetized with 2-phenoxyethanol (500 ppm), and transferred onto glass slides. A few drops of 3% low-melting agarose were laid over the living larvae, which were immediately oriented on the lateral side. The retinas of the embedded larvae were observed using a Zeiss 510 confocal laser scanning microscope (Carl Zeiss AG, Germany). Images were captured at a resolution of 512 × 512 pixels using a 20X (NA 0.75) or 40X (NA 1.2) water immersion objective lens.

To quantify the developmental toxicity of benzimidazole compounds in the zebrafish retina, we measured the shape factor of the IPL in each zebrafish. The shape factor is a parameter that can be analyzed by the Volocity image analysis software package (Perkin-Elmer, Cambridge, MA). If the IPL is a round circle, it is recognized as a long object. Because the shape factor is related to roundness, the shape factor of the long object is low. If the IPL is fragmented, it is recognized as multiple objects. Because the multiple objects are more round than a long object, their shape factor is greater than that of a long object. If multiple objects were recognized in a retinal image, the mean of the shape scores was used for the quantification. The numbers of zebrafish analyzed by the imaging are described in the figure legends.

### Transcriptome analysis of zebrafish eye

Zebrafish treated with 0.3 μM MBZ or BBC from 48 h post-fertilization (hpf) to 60 hpf (12 h exposure), 72 hpf (24 h exposure), and 84 hpf (36 h exposure) were stored in RNAlater (Applied Biosystems, Foster City, CA, USA). The eyes of 50 zebrafish were collected by surgical extraction under a microscope and pooled into one sample. Total RNA from 24 samples (3 exposure times, 2 chemicals, 4 samples per condition) was then extracted using an RNeasy Plus Micro kit (Qiagen, Valencia, CA, USA), qualified by an Agilent Bioanalyzer 2100 (Agilent, Santa Clara, CA, USA), and quantified using a spectrophotometer (NanoDrop ND-100, Wilmington, DE, USA). Three hundred nanograms of total RNA was converted into labeled cRNA using the Low RNA Input Fluorescent Linear Amplification Kit (Agilent). Cy3-labeled cRNA (1.5 μg) was hybridized to Agilent Zebrafish Whole Genome Oligo Microarrays (G2519F) according to the manufacturer's protocol. The hybridized microarrays were scanned (Agilent G2565BA) and analyzed by Feature Extraction software (Agilent). The data were normalized using Agi4x44PreProcess (Lopez-Romero, 2013), a package in Bioconductor (Gentleman et al., 2004). Probes that passed four criteria (gIsSaturated, gIsFeatNonUnifOL, gIsPosAndSignif, gIsWellAboveBG) across the dataset were used for further analysis. RankProd analysis (Breitling et al., 2004) was performed to identify differentially expressed genes (DEGs) between two groups by calculating the false discovery rate (FDR). The lists of DEGs (FDR < 20%) at 12, 24, and 36 h of exposure are shown in Table S1-1, S1-2, and S1-3, respectively. The lists of zebrafish genes were then compared with known human orthologs using the Life Science Knowledge Bank (World Fusion, Tokyo, Japan). In the Life Science Knowledge Bank, the zebrafish gene symbols were mapped to Entrez IDs (Maglott et al., 2011), which were then mapped to human gene symbols. As shown in Table S1, about 55% of zebrafish gene symbols could be mapped to Entrez IDs and about 55% of Entrez IDs could be mapped to human gene symbols. UniProt IDs of the human orthologous genes were added using DAVID (Dennis et al., 2003). The microarray data have been deposited to the Gene Expression Omnibus (GEO) as GSE75245.

### Identification of wikipathways enriched in the DEGs

To identify WikiPathways (Kelder et al., 2012) involved in the developmental toxicity of MBZ, ClueGO (Bindea et al., 2009) and CluePedia (Bindea et al., 2013) in Cytoscape (Shannon et al., 2003) were used. The lists of genes dysregulated in zebrafish eyes exposed to MBZ for 12, 24, and 36 h were subjected to ClueGO with CluePedia using the default settings. The WikiPathways significantly enriched in DEG in zebrafish eyes exposed to MBZ for 12, 24, and 36 h are shown in Table S2-1, S2-2, and S2-3, respectively. The WikiPathways significantly enriched in common among 12, 24, and 36 h exposure are shown in Table S2-4. The *p*-values of each WikiPathway for the enrichment are shown as the size of the circle. WikiPathways clustered in the same group are shown in the same color and connected with lines based on their kappa score (Bindea et al., 2009).

### Identification of biocarta pathways related to the DEGs

To identify BioCarta pathways (Nishimura, 2001) involved in the developmental toxicity of MBZ, JEPETTO (Winterhalter et al., 2014) was used. The genes dysregulated in zebrafish eyes exposed to MBZ for 12, 24, and 36 h were subjected to JEPETTO using the default settings. The BioCarta pathways significantly related to DEGs in zebrafish eyes exposed to MBZ for 12, 24, and 36 h are listed in Table S3-1, S3-2, and S3-3, respectively. The BioCarta pathways related to DEG at 12, 24, and/or 36 h exposure were clustered based on their q-value for relationship using hierarchical clustering with Pearson's correlation (uncentered, average linkage) in MeV (Howe et al., 2011).

### Identification of transcription factors potentially regulating the DEGs

To identify transcription factors (TFs) involved in the developmental toxicity of MBZ, iRegulon (Janky et al., 2014) was used. iRegulon exploits the fact that genes that are co-regulated by the same TF commonly share binding sites for the TF. iRegulon has been successfully used to identify TFs in given gene lists (Nishimura et al., 2015b) using ENCODE ChIP-seq data as a reference database (Janky et al., 2014). The genes dysregulated in zebrafish eyes exposed to MBZ for 12, 24, and 36 h were subjected to iRegulon using the default settings. The predicted TFs for DEGs in zebrafish eyes exposed to MBZ for 12, 24, and 36 h are listed in Table S4-1, S4-2, and S4-3, respectively. The TFs potentially regulating the DEGs in zebrafish eyes exposed to MBZ for 12, 24, and 36 h were clustered based on their normalized enrichment scores using hierarchical clustering with Pearson's correlation (uncentered, average linkage) in MeV (Howe et al., 2011).

### TUNEL staining

TUNEL staining was performed using ApopTag Fluorescein In situ Apoptosis Detection Kit (Millipore, Billerica, MA, USA) according to the manufacturer's protocol. Briefly, zebrafish exposed to either 0.5 μM MBZ or 0.1% DMSO with or without 2 μM KU-55933 from 2 to 3 dpf were fixed in 4% paraformaldehyde in phosphate-buffered saline (PBS; Nacalai Tesque, Kyoto, Japan) at 4°C overnight. Eight zebrafish were used for each conditions except for 0.1% DMSO and 2 μM KU-55933 (*n* = 5). Zebrafish were washed with PBS with 0.1% Tween 20 (PBST) and incubated in water containing 3% H_2_O_2_ and 1% KOH at room temperature for 30 min. Zebrafish were then washed with PBST and incubated with 100% methanol at −30°C overnight. After rehydration, zebrafish were treated with proteinase K (40 μg/mL) at 37°C for 1 h. Zebrafish were washed with PBST, and then incubated in the equilibration buffer at 37°C for 1 h, followed by incubation in the working solution containing TdT enzyme and digoxigenin-labeled dNTP at 37°C for 1 h. Zebrafish were washed and treated with anti-digoxigenin IgG labeled with fluorescein at 4°C overnight. Zebrafish were then washed with PBST again and imaged using the SMZ25 stereomicroscope (Nikon, Tokyo, Japan) with a GFP filter. Quantitative analysis of the fluorescent image was performed using the Volocity software (PerkinElmer, Waltham, MA, USA), with 80 as the threshold of fluorescent intensity of apoptosis. Excluding the lens, the area of fluorescent signal in the retina with a measured intensity over the threshold was deemed an apoptotic signal. The area of zebrafish eye was also measured. The apoptotic signal was normalized by the area of eye in each zebrafish to account for different eye size.

### Statistical analysis

Statistical analysis was performed using Prism 6 (GraphPad, La Jolla, CA, USA). The means were compared by analysis of variance. Alpha was set at 0.05 and Dunnett's multiple comparisons test was used for *post hoc* analyses when significant effects were found. Data are shown as the mean ± SEM.

## Results

### MBZ caused developmental toxicity in the zebrafish retina

**Figure 1:**
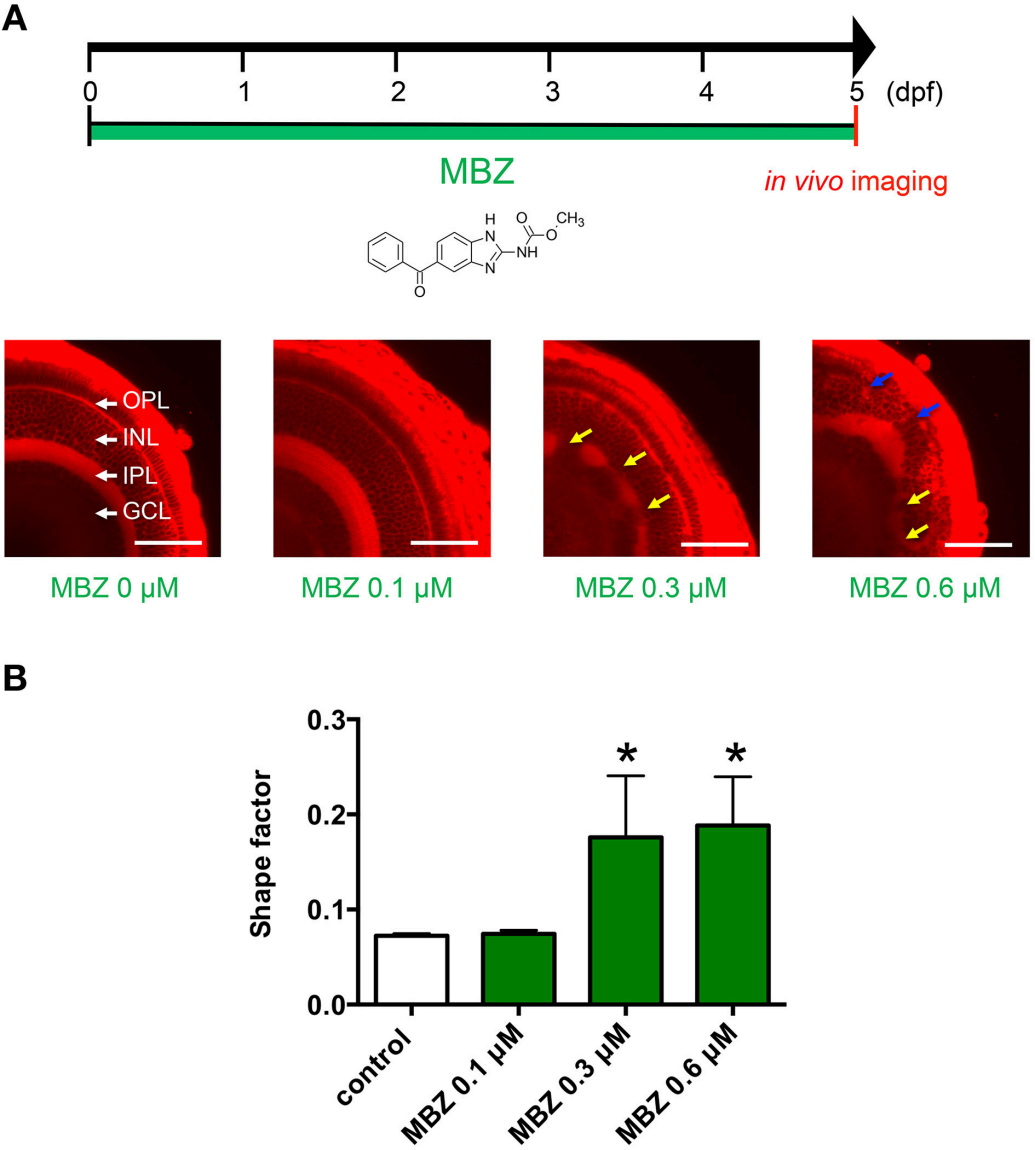
**MBZ caused developmental toxicity in the zebrafish retina**. **(A)** Zebrafish were treated with MBZ from 0 to 5 dpf at the indicated concentration. At 5 dpf, zebrafish were stained with ZMA462 and the retinas were imaged *in vivo*. The GCL, IPL, INL, and OPL are indicated by white arrows. The malformations in the IPL and OPL caused by MBZ are indicated by yellow and blue arrows, respectively. Scale bar: 50 μm. **(B)** Quantitative analysis of the developmental toxicity of MBZ in the zebrafish retina. The shape of the IPL in each zebrafish were quantified using the shape factor. *n* = 8 for control, *n* = 3 for each concentration, ^*^p < 0.05 compared with control.

The dose–response relationship between MBZ and developmental toxicity was examined using *in vivo* imaging of zebrafish retinas stained with the fluorescent dye ZMA462. Figure 1 shows the layers of the zebrafish retina, including the GCL, IPL, INL, and OPL. The layers were clearly visualized at 5 dpf. In zebrafish treated with 0.1 μM MBZ from 0 to 5 dpf, these layers were not different from those of control zebrafish (Figure 1A). In contrast, the IPL in zebrafish treated with 0.3 μM MBZ was malformed, compared with the IPL in control zebrafish (Figure 1A). In zebrafish treated with 0.6 μM MBZ, not only the IPL but also the OPL was severely malformed (Figure 1A). The shape of the IPL in each retina was quantified using the shape factor, as described in the Materials and Methods section. The shape factors of the IPL in zebrafish exposed to 0.3 and 0.6 μM MBZ were significantly greater than that of control zebrafish (Figure 1B). These results suggest that MBZ can cause developmental toxicity in the zebrafish retina when zebrafish are exposed to MBZ at or over 0.3 μM from 0 to 5 dpf.

**Figure 2:**
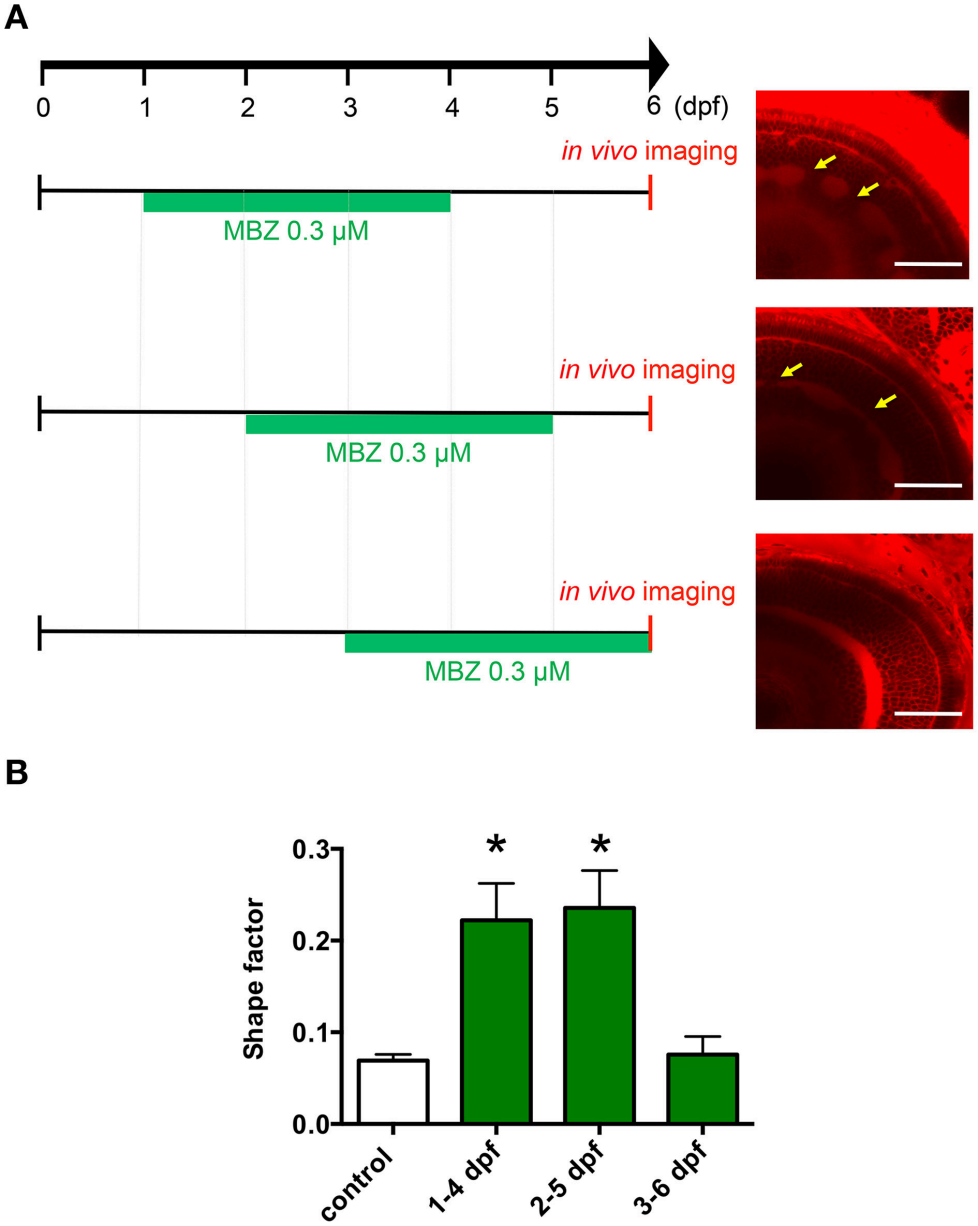
**Development during 2 and 3 dpf was the critical window for developmental toxicity of MBZ in the zebrafish retina. (A)** Zebrafish were treated with 0.3 μM MBZ during the indicated periods. At 6 dpf, zebrafish were stained with ZMA462 and the retinas were imaged *in vivo*. The malformations in the IPL caused by MBZ are indicated by yellow arrows. Scale bar: 50 μm. **(B)** Quantitative analysis of the developmental toxicity of 0.3 μM MBZ in the zebrafish retina. The shape of the IPL in each zebrafish was quantified using the shape factor. *n* = 5 for control, *n* = 6 for 1–4 dpf, *n* = 4 for 2–5 dpf and 3–6 dpf, ^*^p < 0.05 compared with control.

To examine which developmental stage may be susceptible to the toxicity of MBZ in the zebrafish retina, zebrafish were treated with 0.3 μM MBZ from 1 to 4 dpf, 2 to 5 dpf, or 3 to 6 dpf. The *in vivo* imaging of the zebrafish retina at 6 dpf revealed that the IPL was malformed in zebrafish treated with MBZ from 1 to 4 dpf and 2 to 5 dpf, but not 3 to 6 dpf (Figure 2A). The shape factors of the IPL in zebrafish exposed to 0.3 μM MBZ from 1 to 4 dpf or 2 to 5 dpf, but not from 3 to 6 dpf, were significantly greater than that of control zebrafish (Figure 2B). These results suggest that the developmental stage during 2 and 3 dpf is the critical window for developmental toxicity of MBZ in the zebrafish retina.

To examine whether benzimidazole compounds structurally related to MBZ may cause developmental toxicity in the zebrafish retina, zebrafish were treated with 0.03 μM ABZ, 0.15 μM NCZ, or 0.3 μM benzoic acid, BBC. As shown in Figure S1A, both ABZ and NCZ but not BBC caused malformation in the zebrafish retina similar to that induced by MBZ. The shape factors of the IPL in zebrafish exposed to 0.03 μM ABZ or 0.15 μM NCZ, but not 0.3 μM BBC, were significantly greater than that of control zebrafish Figure S1B. These results are consistent with previous reports demonstrating that ABZ and NCZ but not BBC can cause developmental toxicity in zebrafish (Liu and Lessman, 2007; Kitambi et al., 2009; Mattsson et al., 2012).

### DNA damage response was involved in the developmental toxicity of MBZ

To identify the mechanism underlying the developmental toxicity of MBZ in the zebrafish retina, we performed transcriptome analysis of the eyes of zebrafish treated with 0.3 μM MBZ or BBC from 48 hpf to 60 hpf (12 h exposure), 72 hpf (24 h exposure), and 84 hpf (36 h exposure); 41, 50, and 117 genes were identified as DEGs in zebrafish exposed to MBZ, compared with zebrafish exposed to BBC, at 12, 24, and 36 h, respectively Table S1.

**Figure 3:**
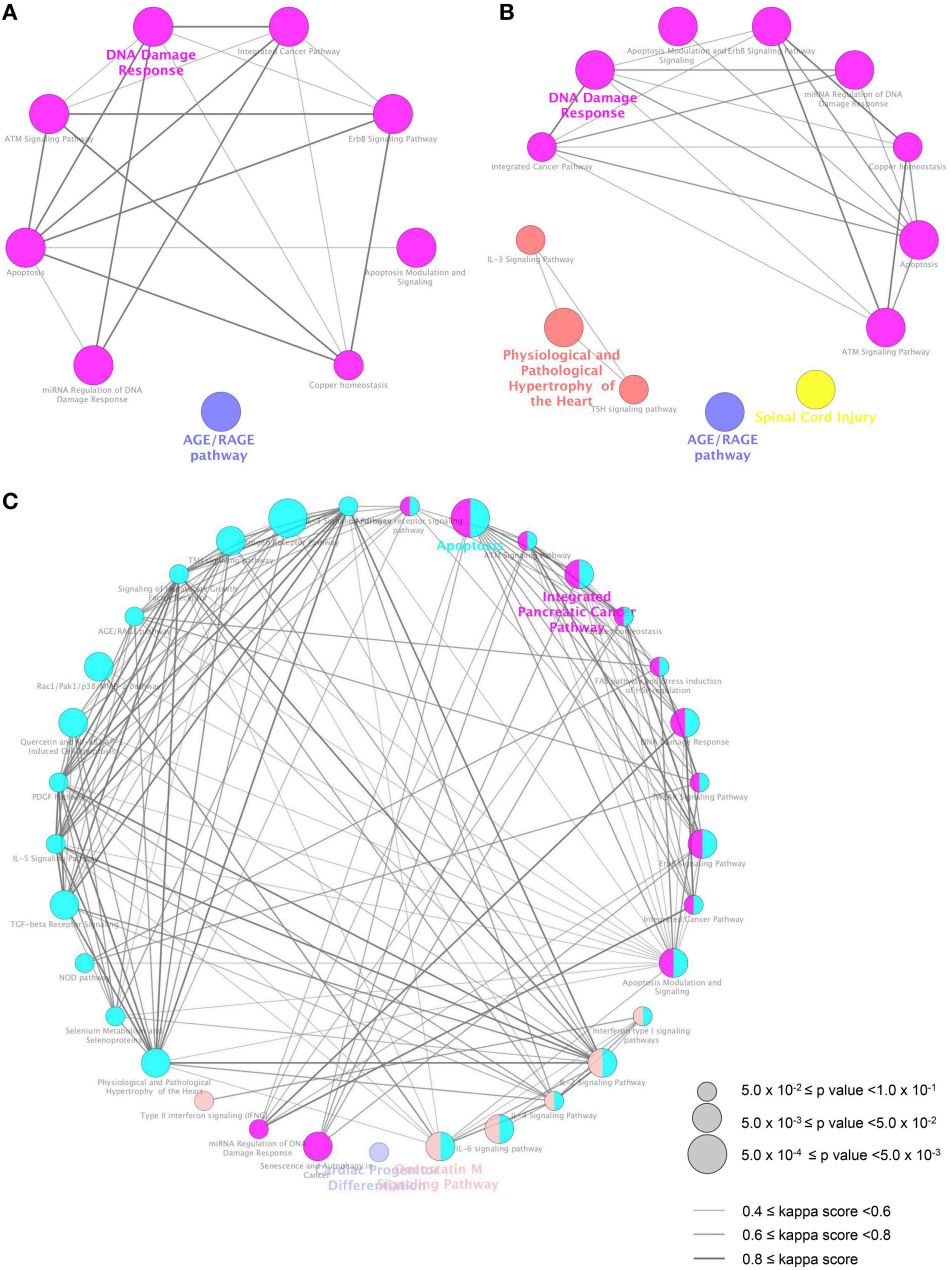
**Pathways significantly enriched in the DEGs in zebrafish exposed to MBZ**. Genes dysregulated in zebrafish exposed to 0.3 μM MBZ for 12, 24, and 36 h were independently subjected to ClueGO using WikiPathways as the database. The pathways significantly enriched in the DEGs at 12, 24, and 36 h exposure are shown in **A**, **B**, and **C**, respectively. Each circle represents one WikiPathway. The size of circle corresponds to the *p* value for the enrichment. Pairs of WikiPathways with a similar kappa score were connected by lines. WikiPathways clustered in a same group are shown in same color.

To elucidate the pathway involved in the genes dysregulated by MBZ, ClueGO (Bindea et al., 2009) was used. ClueGO is a bioinformatics tool that has been used successfully to decipher functionally grouped gene ontology and pathway annotation networks in given lists, combined with various databases such as WikiPathways (Kelder et al., 2012). ClueGO identified 9, 13, and 33 WikiPathways significantly enriched in the DEGs in zebrafish exposed to MBZ for 12, 24, and 36 h, respectively (Figure 3, Table S2-1, S2-2, and S2-3). Among the nine WikiPathways enriched in the DEGs in zebrafish exposed to MBZ for 12 h, eight WikiPathways were clustered into a group where “DNA damage response” was the most significant (Figure 3A). Similarly, among the 13 WikiPathways enriched in the DEGs in zebrafish exposed to MBZ for 24 h, eight WikiPathways were clustered into a group where “DNA damage response” was the most significant (Figure 3B). Among the 33 WikiPathways enriched in the DEGs in zebrafish exposed to MBZ for 36 h, 13 WikiPathways, including “DNA damage response,” were clustered into a group (Figure 3C).

The eight WikiPathways enriched in the DEG in zebrafish exposed to MBZ for 12 h were also enriched in DEGs in zebrafish exposed to MBZ for 24 h and 36 h Tables S2-4. *CASP8, TP53*, and *MDM2* are involved in most of the eight WikiPathways. Expression of these genes was upregulated by MBZ at 12, 24, and 36 h exposure Table S1. It has been shown that DNA damage response activates ATM (Sherman et al., 2011) and that activated ATM induces the expression of *CASP8* (Geiger et al., 2012), *TP53* (Shao et al., 2009), and *MDM2* (Kim and Jackson, 2013). These results suggest that MBZ may cause developmental toxicity in the zebrafish retina by activating the DNA damage response.

### Identification of ATM signaling as a key pathway in the developmental toxicity of MBZ

**Figure 4:**
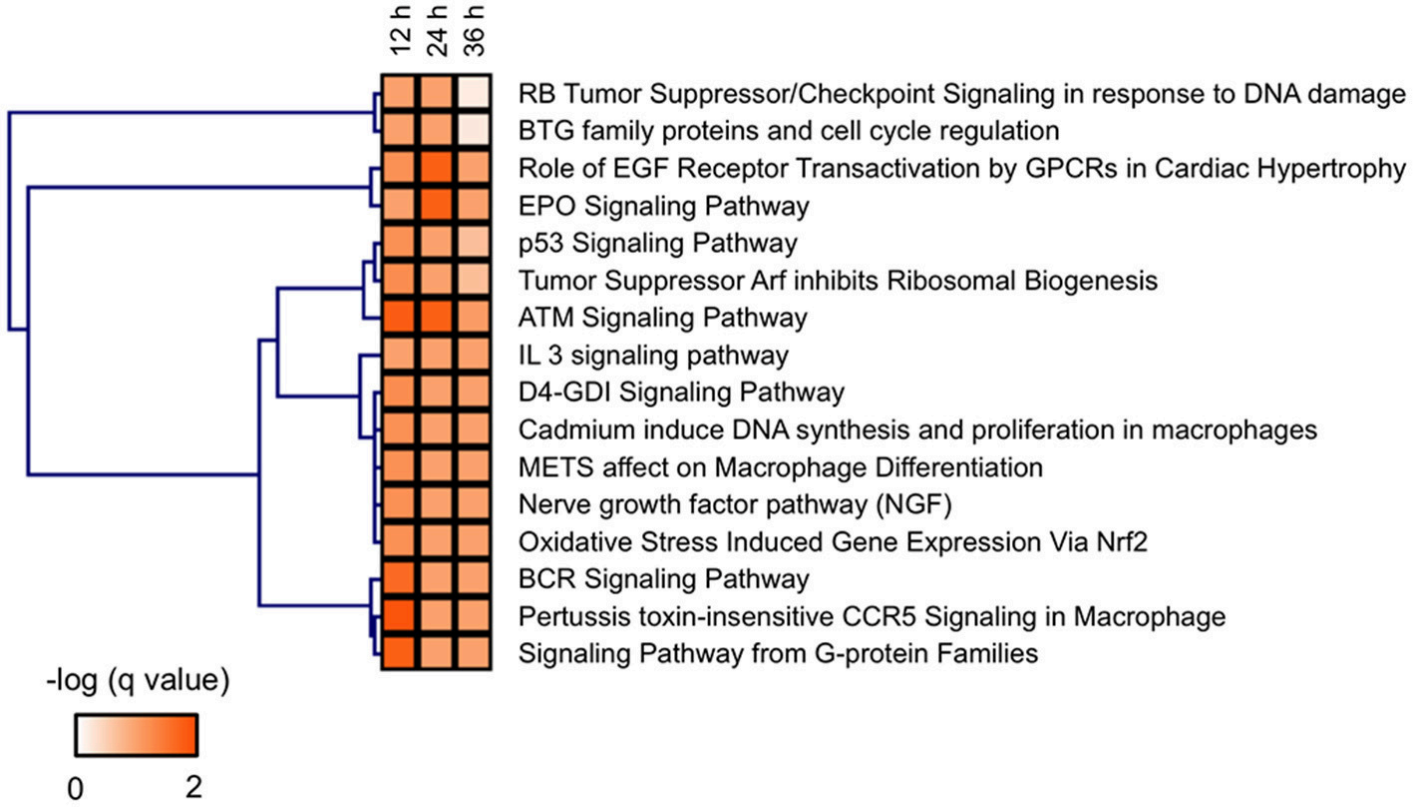
**BioCarta pathways significantly related to the genes dysregulated in zebrafish exposed to MBZ**. Genes dysregulated in zebrafish exposed to 0.3 μM MBZ for 12, 24, and 36 h were independently subjected to JEPETTO using BioCarta as the database. The BioCarta pathways significantly related to the dysregulated genes at each time points were subjected to hierarchical clustering using the q-value for significance.

Functional networks significantly related to the DEGs in zebrafish exposed to MBZ were examined using JEPETTO (Winterhalter et al., 2014), to identify functional associations between genes and pathways using protein interaction networks such as BiocCarta (Nishimura, 2001). JEPETTO identified 12, 10, and 5 functional networks significantly related to the DEGs in zebrafish exposed to MBZ for 12, 24, and 36 h, respectively Table S3-1, S3-2, and S3-3. Figure 4 shows the hierarchical clustering of these functional networks based on the significance. We identified three networks, associated with “ATM signaling pathway,” “p53 signaling pathway,” and “tumor suppressor ARF inhibits ribosomal biogenesis,” in common at 12, 24, and 36 h exposure to MBZ Table S3-4. The ATM signaling pathway was also identified by ClueGO (Figure 3 and Table S2-4), suggesting that the ATM signaling pathway may be a key pathway involved in the developmental toxicity of MBZ. Figure S2 shows the protein interaction networks of the ATM signaling pathway significantly related to the DEGs in zebrafish exposed to MBZ for 12, 24, and 36 h. Among the networks, the expression of *TP53, MDM2, JUN, FOS*, and *CCNG1* was upregulated by exposure to MBZ for 12, 24, and 36 h (Figure S2). These results suggest that MBZ may upregulate the expression of these genes by activating the ATM signaling pathway.

### Identification of ATF2 as a TF potentially involved in the developmental toxicity of MBZ

**Figure 5:**
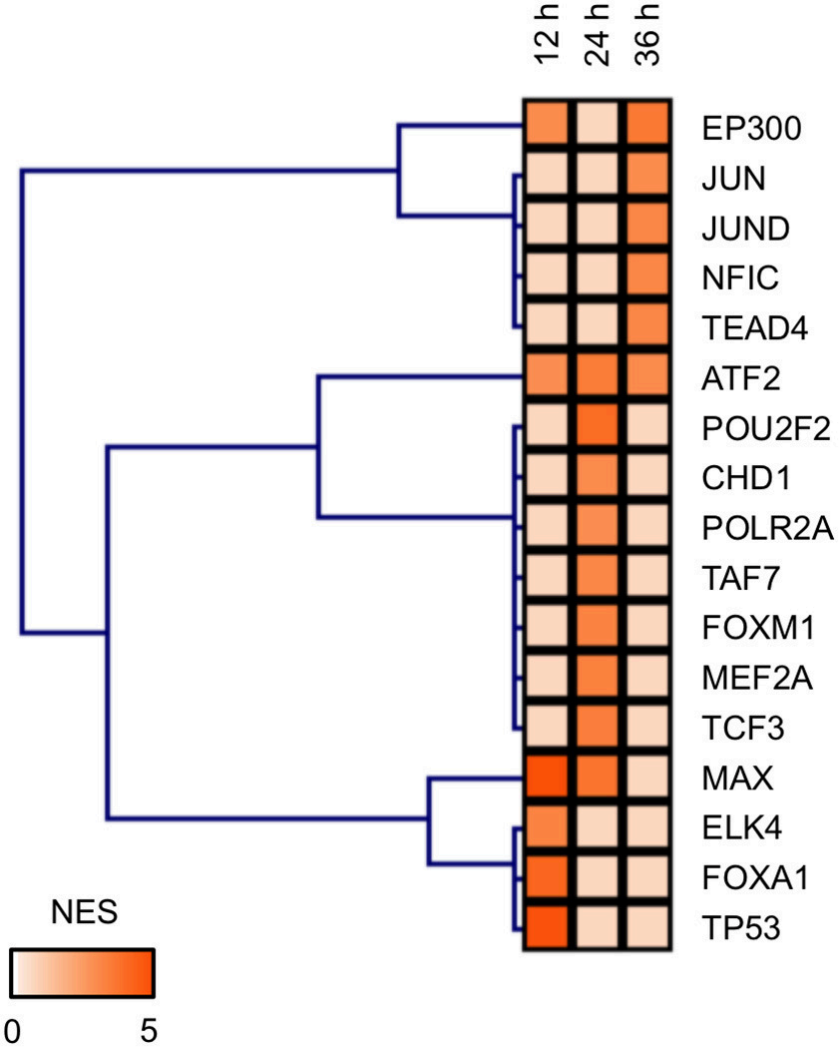
**TFs potentially regulating the differentially expressed genes in zebrafish exposed to MBZ**. Genes dysregulated in zebrafish exposed to 0.3 μM MBZ for 12, 24, and 36 h were independently subjected to iRegulon. The TFs potentially regulating the dysregulated genes at each time point were subjected to hierarchical clustering using the normalized enrichment score (NES) for significance.

TFs potentially regulating the DEGs in zebrafish exposed to MBZ were examined using iRegulon; this program has been used successfully to identify TFs in given gene lists using ENCODE Chip-Seq data (Gerstein et al., 2012; Janky et al., 2014; Nishimura et al., 2015b). iRegulon identified 6, 9, and 6 TFs potentially regulating the genes dysregulated in zebrafish exposed to MBZ for 12, 24, and 36 h, respectively Table S4. Figure 5 shows the hierarchical clustering of these TFs based on the significance. ATF2 is identified in common as the TF potentially regulating these genes. Figure S3 shows the networks between ATF2 and potential target genes at 12, 24, and 36 h exposure to MBZ. Several genes are overlapped among the three networks, including *JUN, FOS*, and *ATF3*. These results are consistent with previous studies demonstrating that *JUN, FOS*, and *ATF3* are transcriptionally activated by ATF2 (Liang et al., 1996; Kristiansen et al., 2010; Lindaman et al., 2013). It has also been demonstrated that ATF2 is activated by ATM through phosphorylation in the DNA damage response (Bhoumik et al., 2005). These results suggest that MBZ may cause developmental toxicity in the zebrafish retina through the activation of ATM-ATF2 signaling during the DNA damage response.

### Inhibition of ATM attenuated the apoptosis induced by MBZ in the zebrafish retina

**Figure 6:**
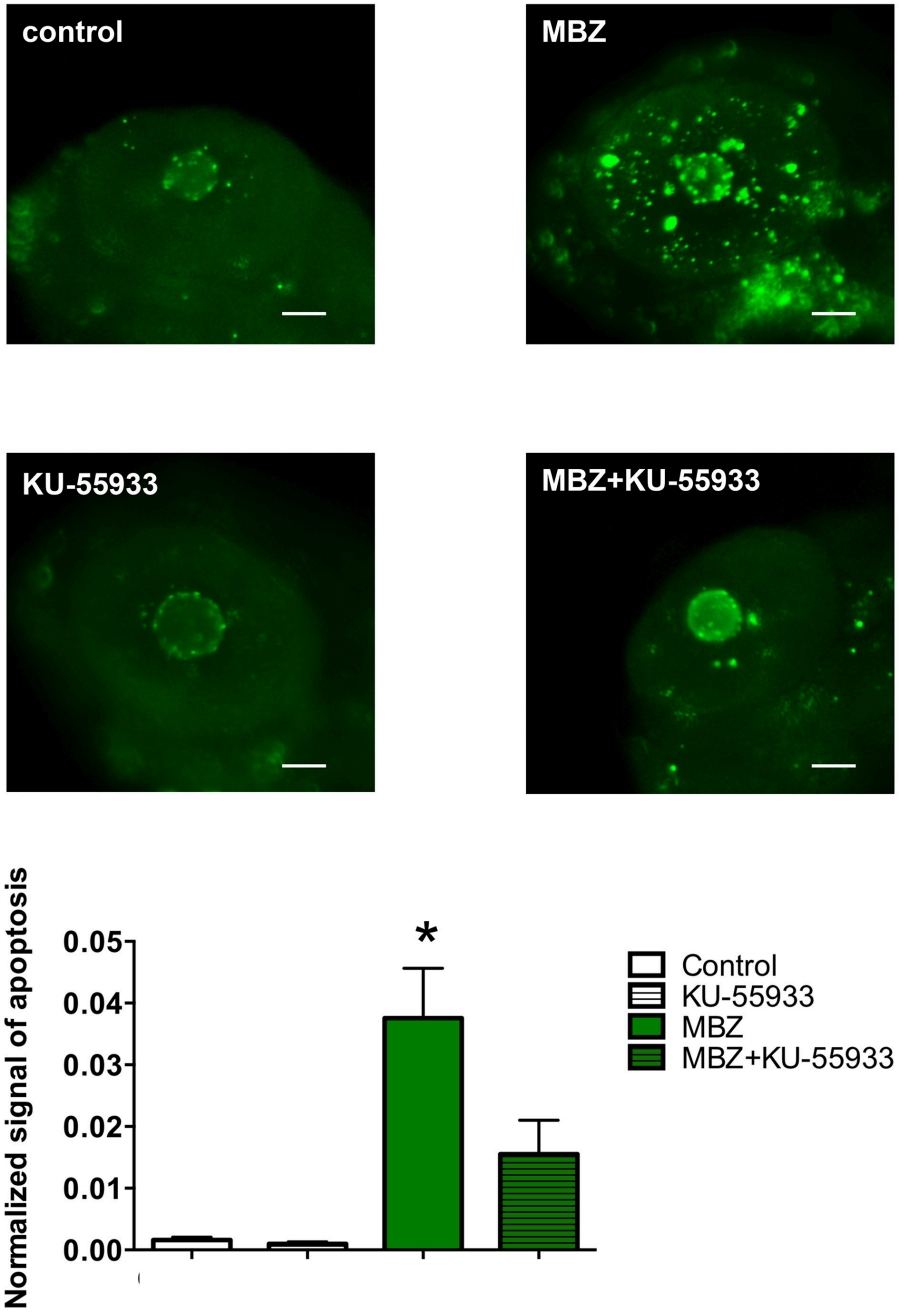
**Inhibition of ATM attenuated apoptosis induced by MBZ in the zebrafish retina**. Zebrafish were exposed to 0.5 μM MBZ or 0.1% DMSO with or without 2 μM KU-55933 from 2 to 3 dpf. After exposure, zebrafish were subjected to TUNEL staining. The areas of apoptotic signal in the retina were measured and normalized by the area of retina. *n* = 8 per each group except for 0.1% DMSO and 2 μM KU-5593 (*n* = 5). ^*^p < 0.05 compared with control. Scale bar: 50 μm.

To examine the involvement of ATM in the developmental toxicity of MBZ in the zebrafish retina, apoptosis in the retina after exposure to MBZ with and without KU-55933, a pharmacological inhibitor of ATM (Hickson et al., 2004), was examined. As shown in Figure 6, apoptosis in the retina of zebrafish exposed to 0.5 μM MBZ from 2 to 3 dpf was significantly increased, compared with that of control zebrafish. In contrast, apoptosis in the retina of zebrafish exposed to 0.5 μM MBZ and 2 μM KU-55933 from 2 to 3 dpf was not significantly different from that of control zebrafish. These results suggest that MBZ can cause developmental toxicity in the zebrafish retina, at least partly through activation of ATM.

## Discussion

### MBZ increases DNA damage response in the developing zebrafish retina

This study demonstrated that the DNA damage response is activated in the retina of zebrafish exposed to MBZ during 2 to 3 dpf. Because the formation of retinal layers in zebrafish occurs during 2 to 3 dpf (Watanabe et al., 2010), the DNA damage response activated by MBZ in zebrafish may affect the differentiation and maturation of retinal cells, including ganglion cells in the GCL, bipolar cells in the INL, and photoreceptor cells in the ONL, that form synapses in the IPL and OPL.

MBZ, ABZ, and NCZ bind to the colchicine-binding domain of tubulin, resulting in the inhibition of tubulin polymerization (Morgan et al., 1993; Xu et al., 2002). Microtubule-targeting agents such as vincristine and paclitaxel can augment the DNA damage response, induced by DNA damaging agents, by disrupting intracellular trafficking of DNA repair proteins (Poruchynsky et al., 2015). The DNA damage response causes not only transient cell cycle arrest coupled with DNA repair but also apoptosis and cell differentiation, especially in neuronal cells (Barzilai et al., 2008; O'driscoll and Jeggo, 2008; Sherman et al., 2011). It has been demonstrated that oxidative DNA damage increases during postnatal development in the mouse retina, causing apoptosis in the INL, which peaked at postnatal day 7 (Martin-Oliva et al., 2015). It has also been shown that apoptosis in the developing zebrafish retina peaks at 3 dpf in the GCL and INL, where retinal ganglion cells and amacrine cells are differentiating during the developmental stage (Biehlmaier et al., 2001). These findings suggest that the DNA damage response may be naturally activated at around 3 dpf in the GCL and INL of the zebrafish retina. In addition, exposure to benzimidazole compounds such as MBZ during this period may overactivate the DNA damage response, resulting in the excessive apoptosis of retinal cells and malformation of the IPL and OPL, where the synapses between retinal ganglion cells and bipolar cells and between bipolar cells and photoreceptor cells are formed. These findings are consistent with previous reports showing the stage-dependent developmental toxicity of benzimidazole compounds in zebrafish (Mattsson et al., 2012; Boix et al., 2015). It is also noteworthy that benzimidazole compounds possess DNA-damaging properties (Locatelli et al., 2004; Giunta et al., 2010; Patel et al., 2011). Altogether, the findings suggest that benzimidazole compounds, such as MBZ, ABZ, and NCZ may overactivate the DNA damage response in zebrafish by disrupting intracellular trafficking of DNA repair proteins and/or causing direct DNA damage during development of the vertebrate retina.

### MBZ activates ATM in the developing zebrafish retina

This study demonstrated that the ATM-ATF2 pathway may be activated in the retina of zebrafish exposed to MBZ, and that the inhibition of ATM significantly attenuated apoptosis in the retina.

ATM is a member of the phosphatidylinositol 3-kinase family that phosphorylates key substrates involved in the DNA damage response (Barzilai et al., 2008; O'driscoll and Jeggo, 2008), and ATF2 is one of the main downstream targets of ATM in the DNA damage response (Lau and Ronai, 2012). Mutation of ATM causes ataxia telangiectasia, a congenital disorder of malformation in various tissues. It has been shown that ATM regulates apoptosis in the developing mouse retina (Rodrigues et al., 2013) and that impairment of the ATM pathway of DNA damage response signaling causes malformation in the eyes of humans with ZNF423 mutation (Chaki et al., 2012). Mutation of *Atf2* also causes malformation in the murine nervous system (Ackermann et al., 2011). These results suggest that the ATM-ATF2 pathway in the vertebrate retina is tightly regulated in a developmentally stage-specific manner, and that perturbation of the ATM-ATF2 pathway may cause developmental toxicity in the vertebrate eye. It has also been demonstrated that NCZ can activate ATM in HeLa cells (Yang et al., 2011). These results suggest that benzimidazole anthelmintics may cause developmental toxicity in the vertebrate retina through overactivation of the ATM-ATF2 pathway.

### Tissue selectivity in developmental toxicity related to the DNA damage response

The DNA damage response is physiologically required for differentiation of cells, especially in neuronal tissues (Sherman et al., 2011). Therefore, the timing and intensity of the DNA damage response during development may be tightly regulated in each tissue, depending on the development stage (Barzilai et al., 2008). Exposure to benzimidazole anthelminthics during a developmental stage which involves active DNA damage response may overactivate the response and disturb cell differentiation, resulting in tissue malformation. It has been shown that hydroxyurea, a clinical drug widely used to treat myeloproliferative diseases and sickle cell anemia, causes developmental toxicity in hindlimb, tail, and neural tube defects in mice, and that the tissue selectivity hydroxyurea's toxicity may be related to the intensity of the DNA damage response (Banh and Hales, 2013). It has also been shown that a deficiency in nibrin, a key factor for repair of DNA damage, leads to ATM-dependent apoptotic cell death in the developing retina but not other tissues in mice (Rodrigues et al., 2013), suggesting that the retina may be highly sensitive to DNA damage. Furthermore, it has been demonstrated that MBZ activates the MEK/ERK pathway (Mizuno et al., 2011) and that ERK facilitates activation of ATM during the DNA damage response (Wei et al., 2011). Consistent with these findings, inhibition of MEK/ERK signaling by U0126 significantly attenuated apoptosis in the retina of zebrafish exposed to MBZ (data not shown). Altogether, these findings suggest that chemicals that can activate both the DNA damage response and MEK/ERK pathway may cause developmental toxicity in the mammalian retina.

In summary, by using a systematic toxicology approach, this study demonstrated that overactivation of the DNA damage response, including the ATM signaling pathway, is involved in the developmental toxicity of MBZ to the zebrafish retina. More studies are required to examine whether overactivation of the DNA damage response is also involved in the developmental toxicity of benzimidazole anthelmintics in the mammalian retina, and other tissues sensitive to DNA damage.

## Author contributions

YN conceived the study, performed bioinformatics analyses, and wrote the manuscript. SS performed the experiments to validate the effects of ATM and the MEK/ERK pathway and analyzed the data. TK performed *in vivo* imaging of zebrafish retina and the transcriptome analysis. YY, SM, YA, MY, SO, KK, and RK performed experiments. TT conceived the study and wrote the manuscript.

### Conflict of interest statement

The authors declare that the research was conducted in the absence of any commercial or financial relationships that could be construed as a potential conflict of interest.

## Supplementary material

Figure S1 **ABZ and NCZ but not BBC caused developmental toxicity in the zebrafish retina. (A)** Zebrafish were treated with 0.03 μM ABZ, 0.15 μM NCZ, or 0.3 μM BBC from 2 to 5 dpf at the indicated concentration. Zebrafish were stained with ZMA462 and the retinas were imaged *in vivo*. Scale bar: 50 μm. **(B)** Quantitative analysis of the developmental toxicity of benzimidazole compounds in the zebrafish retina. The shape of the IPL in each zebrafish was quantified using the shape factor. *n* = 8 for control, *n* = 4 for ABZ, *n* = 3 for NCZ and BBC, ^*^*p* < 0.05 compared with control.

Figure S2 **Protein interaction networks associated with the ATM signaling pathway at 12, 24, and 36 h exposure to 0.3 μM MBZ**. The protein interaction networks associated with the ATM signaling pathway at 12, 24, and 36 h exposure to 0.3 μM MBZ are shown in **A**, **B**, and **C**, respectively.

Figure S3 **Identification of ATF2 as a TF potentially regulating the DEGs in zebrafish exposed to 0.3 μM MBZ for 12, 24, or 36 h**. The networks between ATF2 and target genes at 12, 24, and 36 h exposure to 0.3 μM MBZ are shown in **A**, **B** and **C**, respectively.

Table S1 **Genes dysregulated in zebrafish exposed to 0.3 μM MBZ. (S1-1)** 12 h exposure; **(S1-2)** 24 h exposure; **(S1-3)** 36 h exposure.

Table S2 **Pathways significantly enriched in the genes dysregulated in zebrafish exposed to 0.3 μM MBZ. (S2-1)** 12 h exposure; **(S2-2)** 24 h exposure; **(S2-3)** 36 h exposure; **(S2-4)** Common pathways significantly enriched in the genes dysregulated in zebrafish 549 exposed to MBZ for 12, 24, and 36 h.

Table S3 **BioCarta pathways significantly related to the genes dysregulated in zebrafish exposed to 0.3 μM MBZ. (S3-1)** 12 h exposure; **(S3-2)** 24 h exposure; **(S3-3)** 36 h exposure; **(S3-4)** Common BioCarta pathways significantly related to the genes dysregulated in zebrafish 557 exposed to MBZ for 12, 24, and 36 h.

Table S4 **TFs potentially regulating the differentially expressed genes in zebrafish exposed to 0.3 μM MBZ. (S4-1)** 12 h exposure; **(S4-2)** 24 h exposure; **(S4-3)** 36 h exposure.

## Abbreviations

MBZ: mebendazole
ABZ: albendazole
NCZ: nocodazole
dpf: days post-fertilization
hpf: hours post-fertilization
DEGs: differentially expressed genes
FDR: false discovery rate
TFs: transcription factors
IPL: inner plexiform layer
OPL: outer plexiform layer
GCL: ganglion cell layer
INL: inner nuclear layer
ONL: outer nuclear layer
DEGs: differentially expressed genes.

## References

[B1] Ackermann J. Ashton G. Lyons S. James D. Hornung J. P. Jones N.. (2011). Loss of ATF2 function leads to cranial motoneuron degeneration during embryonic mouse development. PLoS ONE 6:e19090. 10.1371/journal.pone.0019090 21533046 PMC3080913

[B2] Bal-Price A. K. Coecke S. Costa L. Crofton K. M. Fritsche E. Goldberg A.. (2012). Advancing the science of developmental neurotoxicity (DNT): testing for better safety evaluation. ALTEX 29, 202–215. 10.14573/altex.2012.2.202 22892558

[B3] Banh S. Hales B. F. (2013). Hydroxyurea exposure triggers tissue-specific activation of p38 mitogen-activated protein kinase signaling and the DNA damage response in organogenesis-stage mouse embryos. Toxicol. Sci. 133, 298–308. 10.1093/toxsci/kft069 23492809 PMC3663560

[B4] Barzilai A. Biton S. Shiloh Y. (2008). The role of the DNA damage response in neuronal development, organization and maintenance. DNA Repair (Amst). 7, 1010–1027. 10.1016/j.dnarep.2008.03.005 18458000

[B5] Behl M. Hsieh J. H. Shafer T. J. Mundy W. R. Rice J. R. Boyd W. A. (2015). Use of alternative assays to identify and prioritize organophosphorus flame retardants for potential developmental and neurotoxicity. Neurotoxicol. Teratol. 52(PtB), 181–193. 10.1016/j.ntt.2015.09.003 26386178

[B6] Bhoumik A. Takahashi S. Breitweiser W. Shiloh Y. Jones N. Ronai Z. (2005). ATM-dependent phosphorylation of ATF2 is required for the DNA damage response. Mol. Cell 18, 577–587. 10.1016/j.molcel.2005.04.015 15916964 PMC2954254

[B7] Biehlmaier O. Neuhauss S. C. Kohler K. (2001). Onset and time course of apoptosis in the developing zebrafish retina. Cell Tissue Res. 306, 199–207. 10.1007/s004410100447 11702231

[B8] Bindea G. Galon J. Mlecnik B. (2013). CluePedia Cytoscape plugin: pathway insights using integrated experimental and *in silico* data. Bioinformatics 29, 661–663. 10.1093/bioinformatics/btt019 23325622 PMC3582273

[B9] Bindea G. Mlecnik B. Hackl H. Charoentong P. Tosolini M. Kirilovsky A.. (2009). ClueGO: a Cytoscape plug-in to decipher functionally grouped gene ontology and pathway annotation networks. Bioinformatics 25, 1091–1093. 10.1093/bioinformatics/btp101 19237447 PMC2666812

[B10] Boix N. Teixido E. Vila-Cejudo M. Ortiz P. Ibanez E. Llobet J. M.. (2015). Triclabendazole sulfoxide causes stage-dependent embryolethality in zebrafish and mouse *in vitro*. PLoS ONE 10:e0121308. 10.1371/journal.pone.0121308 25793498 PMC4368200

[B11] Breitling R. Armengaud P. Amtmann A. Herzyk P. (2004). Rank products: a simple, yet powerful, new method to detect differentially regulated genes in replicated microarray experiments. FEBS Lett. 573, 83–92. 10.1016/j.febslet.2004.07.055 15327980

[B12] Carlsson G. Patring J. Kreuger J. Norrgren L. Oskarsson A. (2013). Toxicity of 15 veterinary pharmaceuticals in zebrafish (Danio rerio) embryos. Aquat. Toxicol. 126, 30–41. 10.1016/j.aquatox.2012.10.008 23142600

[B13] Carlsson G. Patring J. Ulleras E. Oskarsson A. (2011). Developmental toxicity of albendazole and its three main metabolites in zebrafish embryos. Reprod. Toxicol. 32, 129–137. 10.1016/j.reprotox.2011.05.015 21683134

[B14] Chaki M. Airik R. Ghosh A. K. Giles R. H. Chen R. Slaats G. G.. (2012). Exome capture reveals ZNF423 and CEP164 mutations, linking renal ciliopathies to DNA damage response signaling. Cell 150, 533–548. 10.1016/j.cell.2012.06.028 22863007 PMC3433835

[B15] Dayan A. D. (2003). Albendazole, mebendazole and praziquantel. Review of non-clinical toxicity and pharmacokinetics. Acta Trop. 86, 141–159. 10.1016/S0001-706X(03)00031-7 12745134

[B16] Dennis G. Jr. Sherman B. T. Hosack D. A. Yang J. Gao W. Lane H. C.. (2003). DAVID: database for annotation, visualization, and integrated discovery. Genome Biol. 4:P3. 10.1186/gb-2003-4-5-p3 12734009 PMC193660

[B17] Geiger K. Hagenbuchner J. Rupp M. Fiegl H. Sergi C. Meister B.. (2012). FOXO3/FKHRL1 is activated by 5-aza-2-deoxycytidine and induces silenced caspase-8 in neuroblastoma. Mol. Biol. Cell 23, 2226–2234. 10.1091/mbc.E11-06-0535 22493319 PMC3364184

[B18] Gentleman R. C. Carey V. J. Bates D. M. Bolstad B. Dettling M. Dudoit S.. (2004). Bioconductor: open software development for computational biology and bioinformatics. Genome Biol. 5:R80. 10.1186/gb-2004-5-10-r80 15461798 PMC545600

[B19] Gerstein M. B. Kundaje A. Hariharan M. Landt S. G. Yan K. K. Cheng C.. (2012). Architecture of the human regulatory network derived from ENCODE data. Nature 489, 91–100. 10.1038/nature11245 22955619 PMC4154057

[B20] Giunta S. Belotserkovskaya R. Jackson S. P. (2010). DNA damage signaling in response to double-strand breaks during mitosis. J. Cell Biol. 190, 197–207. 10.1083/jcb.200911156 20660628 PMC2930281

[B21] Hickson I. Zhao Y. Richardson C. J. Green S. J. Martin N. M. Orr A. I.. (2004). Identification and characterization of a novel and specific inhibitor of the ataxia-telangiectasia mutated kinase ATM. Cancer Res. 64, 9152–9159. 10.1158/0008-5472.CAN-04-2727 15604286

[B22] Howe E. A. Sinha R. Schlauch D. Quackenbush J. (2011). RNA-Seq analysis in MeV. Bioinformatics 27, 3209–3210. 10.1093/bioinformatics/btr490 21976420 PMC3208390

[B23] Janky R. Verfaillie A. Imrichova H. Van De Sande B. Standaert L. Christiaens V.. (2014). iRegulon: from a gene list to a gene regulatory network using large motif and track collections. PLoS Comput. Biol. 10:e1003731. 10.1371/journal.pcbi.1003731 25058159 PMC4109854

[B24] Kelder T. Van Iersel M. P. Hanspers K. Kutmon M. Conklin B. R. Evelo C. T.. (2012). WikiPathways: building research communities on biological pathways. Nucleic Acids Res. 40, D1301–D1307. 10.1093/nar/gkr1074 22096230 PMC3245032

[B25] Kelsh R. N. Brand M. Jiang Y. J. Heisenberg C. P. Lin S. Haffter P.. (1996). Zebrafish pigmentation mutations and the processes of neural crest development. Development 123, 369–389. 9007256 10.1242/dev.123.1.369

[B26] Kim J. K. Jackson T. L. (2013). Mechanisms that enhance sustainability of p53 pulses. PLoS ONE 8:e65242. 10.1371/journal.pone.0065242 23755198 PMC3670918

[B27] Kitambi S. S. Mcculloch K. J. Peterson R. T. Malicki J. J. (2009). Small molecule screen for compounds that affect vascular development in the zebrafish retina. Mech. Dev. 126, 464–477. 10.1016/j.mod.2009.01.002 19445054 PMC2775549

[B28] Kristiansen M. Hughes R. Patel P. Jacques T. S. Clark A. R. Ham J. (2010). Mkp1 is a c-Jun target gene that antagonizes JNK-dependent apoptosis in sympathetic neurons. J. Neurosci. 30, 10820–10832. 10.1523/JNEUROSCI.2824-10.2010 20702711 PMC3044878

[B29] Lau E. Ronai Z. A. (2012). ATF2 - at the crossroad of nuclear and cytosolic functions. J. Cell Sci. 125, 2815–2824. 10.1242/jcs.095000 22685333 PMC3434827

[B30] Law R. Bozzo P. Koren G. Einarson A. (2010). FDA pregnancy risk categories and the CPS: do they help or are they a hindrance? Can. Fam. Physician 56, 239–241. 20228306 PMC2837687

[B31] Liang G. Wolfgang C. D. Chen B. P. Chen T. H. Hai T. (1996). ATF3 gene. Genomic organization, promoter, and regulation. J. Biol. Chem. 271, 1695–1701. 10.1074/jbc.271.3.1695 8576171

[B32] Lindaman L. L. Yeh D. M. Xie C. Breen K. M. Coss D. (2013). Phosphorylation of ATF2 and interaction with NFY induces c-Jun in the gonadotrope. Mol. Cell. Endocrinol. 365, 316–326. 10.1016/j.mce.2012.11.012 23178797 PMC3529762

[B33] Liu J. Lessman C. A. (2007). Soluble tubulin complexes, gamma-tubulin, and their changing distribution in the zebrafish (Danio rerio) ovary, oocyte and embryo. Comp. Biochem. Physiol. B. Biochem. Mol. Biol. 147, 56–73. 10.1016/j.cbpb.2006.12.014 17293149

[B34] Locatelli C. Pedrosa R. C. De Bem A. F. Creczynski-Pasa T. B. Cordova C. A. Wilhelm-Filho D. (2004). A comparative study of albendazole and mebendazole-induced, time-dependent oxidative stress. Redox Rep. 9, 89–95. 10.1179/135100004225004751 15231063

[B35] Lopez-Romero P. (2013). Agi4x44PreProcess: PreProcessing of Agilent 4x44 Array Data. R Package Version 1.20.20. Madrid.

[B36] Maglott D. Ostell J. Pruitt K. D. Tatusova T. (2011). Entrez Gene: gene-centered information at NCBI. Nucleic Acids Res. 39, D52–D57. 10.1093/nar/gkq1237 21115458 PMC3013746

[B37] Martin-Oliva D. Martin-Guerrero S. M. Matia-Gonzalez A. M. Ferrer-Martin R. M. Martin-Estebane M. Carrasco M. C.. (2015). DNA damage, poly(ADP-Ribose) polymerase activation, and phosphorylated histone H2AX expression during postnatal retina development in C57BL/6 mouse. Invest. Ophthalmol. Vis. Sci. 56, 1301–1309. 10.1167/iovs.14-15828 25650421

[B38] Mattsson A. Ulleras E. Patring J. Oskarsson A. (2012). Albendazole causes stage-dependent developmental toxicity and is deactivated by a mammalian metabolization system in a modified zebrafish embryotoxicity test. Reprod. Toxicol. 34, 31–42. 10.1016/j.reprotox.2012.02.007 22414603

[B39] Mizuno K. Toyoda Y. Fukami T. Nakajima M. Yokoi T. (2011). Stimulation of pro-inflammatory responses by mebendazole in human monocytic THP-1 cells through an ERK signaling pathway. Arch. Toxicol. 85, 199–207. 10.1007/s00204-010-0584-y 20848085

[B40] Morgan U. M. Reynoldson J. A. Thompson R. C. (1993). Activities of several benzimidazoles and tubulin inhibitors against Giardia spp. in vitro. Antimicrob. Agents Chemother. 37, 328–331. 10.1128/AAC.37.2.328 8452365 PMC187662

[B41] Mpairwe H. Tweyongyere R. Elliott A. (2014). Pregnancy and helminth infections. Parasite Immunol. 36, 328–337. 10.1111/pim.12101 24471654 PMC4260141

[B42] Nishimura D. (2001). BioCarta. Biotech. Software Internet Rep. 2, 117–120. 10.1089/152791601750294344

[B43] Nishimura Y. Inoue A. Sasagawa S. Koiwa J. Kawaguchi K. Kawase R.. (2016). Using zebrafish in systems toxicology for developmental toxicity testing. Congenit. Anom. (Kyoto). 56, 18–27. 10.1111/cga.12142 26537640

[B44] Nishimura Y. Murakami S. Ashikawa Y. Sasagawa S. Umemoto N. Shimada Y.. (2015a). Zebrafish as a systems toxicology model for developmental neurotoxicity testing. Congenit. Anom. (Kyoto). 55, 1–16. 10.1111/cga.12079 25109898

[B45] Nishimura Y. Sasagawa S. Ariyoshi M. Ichikawa S. Shimada Y. Kawaguchi K.. (2015b). Systems pharmacology of adiposity reveals inhibition of EP300 as a common therapeutic mechanism of caloric restriction and resveratrol for obesity. Front. Pharmacol. 6:199. 10.3389/fphar.2015.00199 26441656 PMC4569862

[B46] Nishimura Y. Yata K. Nomoto T. Ogiwara T. Watanabe K. Shintou T.. (2013). Identification of a novel indoline derivative for *in vivo* fluorescent imaging of blood-brain barrier disruption in animal models. ACS Chem. Neurosci. 4, 1183–1193. 10.1021/cn400010t 23668665 PMC3750685

[B47] O'driscoll M. Jeggo P. A. (2008). The role of the DNA damage response pathways in brain development and microcephaly: insight from human disorders. DNA Repair (Amst). 7, 1039–1050. 10.1016/j.dnarep.2008.03.018 18458003

[B48] Patel K. Doudican N. A. Schiff P. B. Orlow S. J. (2011). Albendazole sensitizes cancer cells to ionizing radiation. Radiat. Oncol. 6:160. 10.1186/1748-717X-6-160 22094106 PMC3231941

[B49] Poruchynsky M. S. Komlodi-Pasztor E. Trostel S. Wilkerson J. Regairaz M. Pommier Y.. (2015). Microtubule-targeting agents augment the toxicity of DNA-damaging agents by disrupting intracellular trafficking of DNA repair proteins. Proc. Natl. Acad. Sci. U.S.A. 112, 1571–1576. 10.1073/pnas.1416418112 25605897 PMC4321245

[B50] Rodrigues P. M. Grigaravicius P. Remus M. Cavalheiro G. R. Gomes A. L. Rocha-Martins M.. (2013). Nbn and atm cooperate in a tissue and developmental stage-specific manner to prevent double strand breaks and apoptosis in developing brain and eye. PLoS ONE 8:e69209. 10.1371/journal.pone.0069209 23935957 PMC3728324

[B51] Salam R. A. Haider B. A. Humayun Q. Bhutta Z. A. (2015). Effect of administration of antihelminthics for soil-transmitted helminths during pregnancy. Cochrane Database Syst. Rev. 6:CD005547. 10.1002/14651858.CD005547.pub3 26087057

[B52] Sasagawa S. Nishimura Y. Koiwa J. Nomoto T. Shinto T. Murakami S.. (2016). *In vivo* detection of mitochondrial dysfunction induced by clinical drugs and disease-associated genes using a novel dye ZMJ214 in zebrafish. ACS Chem. Biol. 11, 381–388. 10.1021/acschembio.5b00751 26630578

[B53] Shannon P. Markiel A. Ozier O. Baliga N. S. Wang J. T. Ramage D.. (2003). Cytoscape: a software environment for integrated models of biomolecular interaction networks. Genome Res. 13, 2498–2504. 10.1101/gr.1239303 14597658 PMC403769

[B54] Shao L. Fujii H. Colmegna I. Oishi H. Goronzy J. J. Weyand C. M. (2009). Deficiency of the DNA repair enzyme ATM in rheumatoid arthritis. J. Exp. Med. 206, 1435–1449. 10.1084/jem.20082251 19451263 PMC2715066

[B55] Sherman M. H. Bassing C. H. Teitell M. A. (2011). Regulation of cell differentiation by the DNA damage response. Trends Cell Biol. 21, 312–319. 10.1016/j.tcb.2011.01.004 21354798 PMC3089693

[B56] Sipes N. S. Padilla S. Knudsen T. B. (2011). Zebrafish: as an integrative model for twenty-first century toxicity testing. Birth Defects Res. C Embryo Today 93, 256–267. 10.1002/bdrc.20214 21932434

[B57] Sturla S. J. Boobis A. R. Fitzgerald R. E. Hoeng J. Kavlock R. J. Schirmer K.. (2014). Systems toxicology: from basic research to risk assessment. Chem. Res. Toxicol. 27, 314–329. 10.1021/tx400410s 24446777 PMC3964730

[B58] Varga Z. M. (2011). Aquaculture and husbandry at the zebrafish international resource center. Methods Cell Biol. 104, 453–478. 10.1016/B978-0-12-374814-0.00024-0 21924177

[B59] Watanabe K. Nishimura Y. Nomoto T. Umemoto N. Zhang Z. Zhang B.. (2012). *In vivo* assessment of the permeability of the blood-brain barrier and blood-retinal barrier to fluorescent indoline derivatives in zebrafish. BMC Neurosci. 13:101. 10.1186/1471-2202-13-101 22894547 PMC3807752

[B60] Watanabe K. Nishimura Y. Oka T. Nomoto T. Kon T. Shintou T.. (2010). *In vivo* imaging of zebrafish retinal cells using fluorescent coumarin derivatives. BMC Neurosci. 11:116. 10.1186/1471-2202-11-116 20843315 PMC2945357

[B61] Wei F. Yan J. Tang D. (2011). Extracellular signal-regulated kinases modulate DNA damage response - a contributing factor to using MEK inhibitors in cancer therapy. Curr. Med. Chem. 18, 5476–5482. 10.2174/092986711798194388 22087839 PMC3330700

[B62] Westerfield M. (2007). A Guide for the Laboratory Use of Zebrafish (Danio rerio). Eugene: University of Oregon Press.

[B63] Whittaker S. G. Faustman E. M. (1992). Effects of benzimidazole analogs on cultures of differentiating rodent embryonic cells. Toxicol. Appl. Pharmacol. 113, 144–151. 10.1016/0041-008X(92)90019-O 1553749

[B64] Winterhalter C. Widera P. Krasnogor N. (2014). JEPETTO: a Cytoscape plugin for gene set enrichment and topological analysis based on interaction networks. Bioinformatics 30, 1029–1030. 10.1093/bioinformatics/btt732 24363376 PMC3967109

[B65] Xu K. Schwarz P. Ludueña R. (2002). Interaction of Nocodazole With Tubulin Isotypes. Drug Dev. Res. 55, 91–96. 10.1002/ddr.10023

[B66] Yang C. Tang X. Guo X. Niikura Y. Kitagawa K. Cui K.. (2011). Aurora-B mediated ATM serine 1403 phosphorylation is required for mitotic ATM activation and the spindle checkpoint. Mol. Cell 44, 597–608. 10.1016/j.molcel.2011.09.016 22099307 PMC3228519

