# Supplementary figures and images for: DNA Damage Response Is Involved in the Developmental Toxicity of Mebendazole in Zebrafish Retina
Source: Front Pharmacol. 2016 Mar 14;7:57. doi: 10.3389/fphar.2016.00057 (PMC4789406; doi:10.3389/fphar.2016.00057)

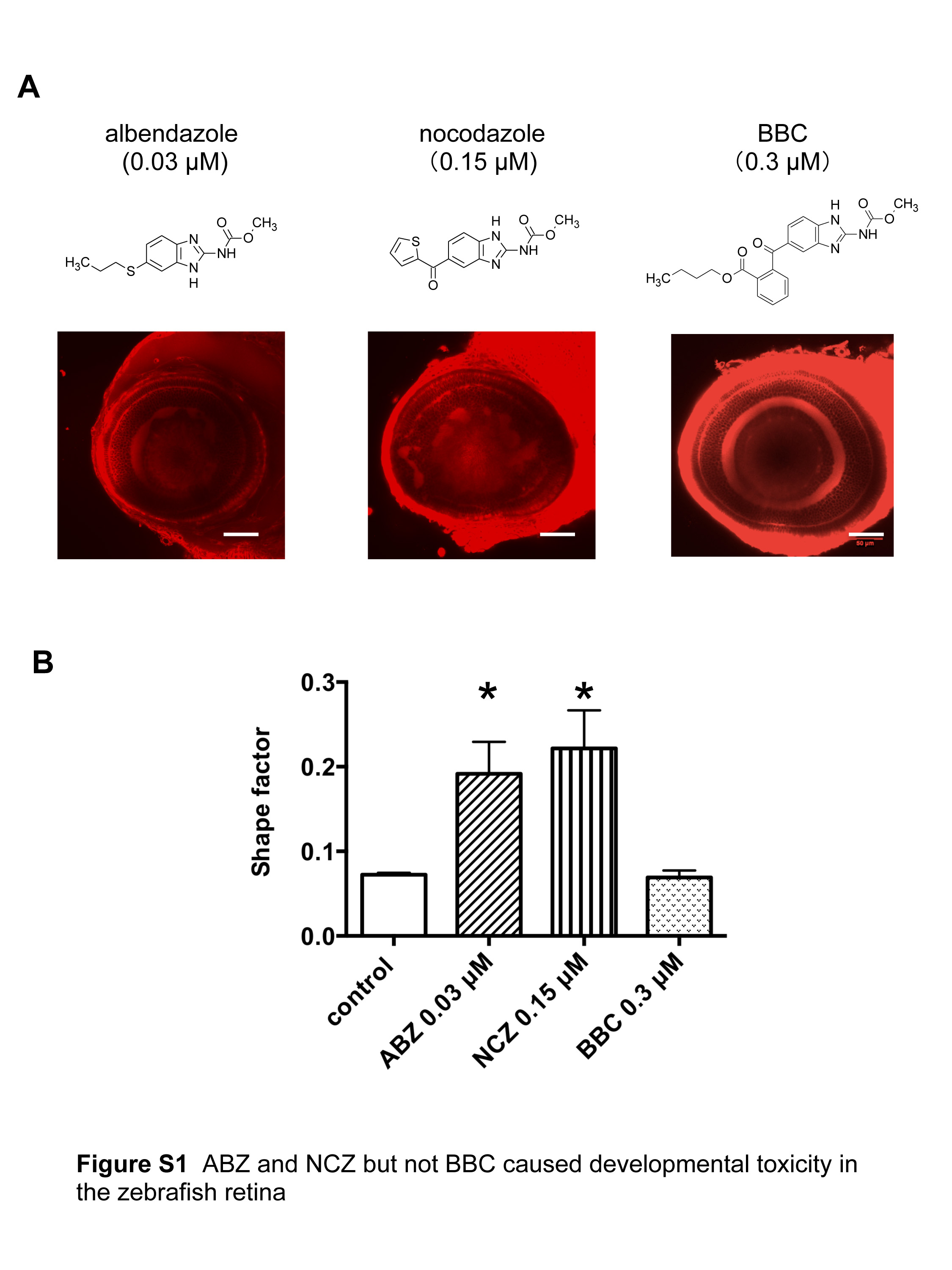

Supplement: Figure S1 — ABZ and NCZ but not BBC caused developmental toxicity in the zebrafish retina. (A) Zebrafish were treated with 0.03 μM ABZ, 0.15 μM NCZ, or 0.3 μM BBC from 2 to 5 dpf at the indicated concentration. Zebrafish were stained with ZMA462 and the retinas were imaged in vivo. Scale bar: 50 μm. (B) Quantitative analysis of the developmental toxicity of benzimidazole compounds in the zebrafish retina. The shape of the IPL in each zebrafish was quantified using the shape factor. n = 8 for control, n = 4 for ABZ, n = 3 for NCZ and BBC, *p < 0.05 compared with control. [file Image1.jpg]

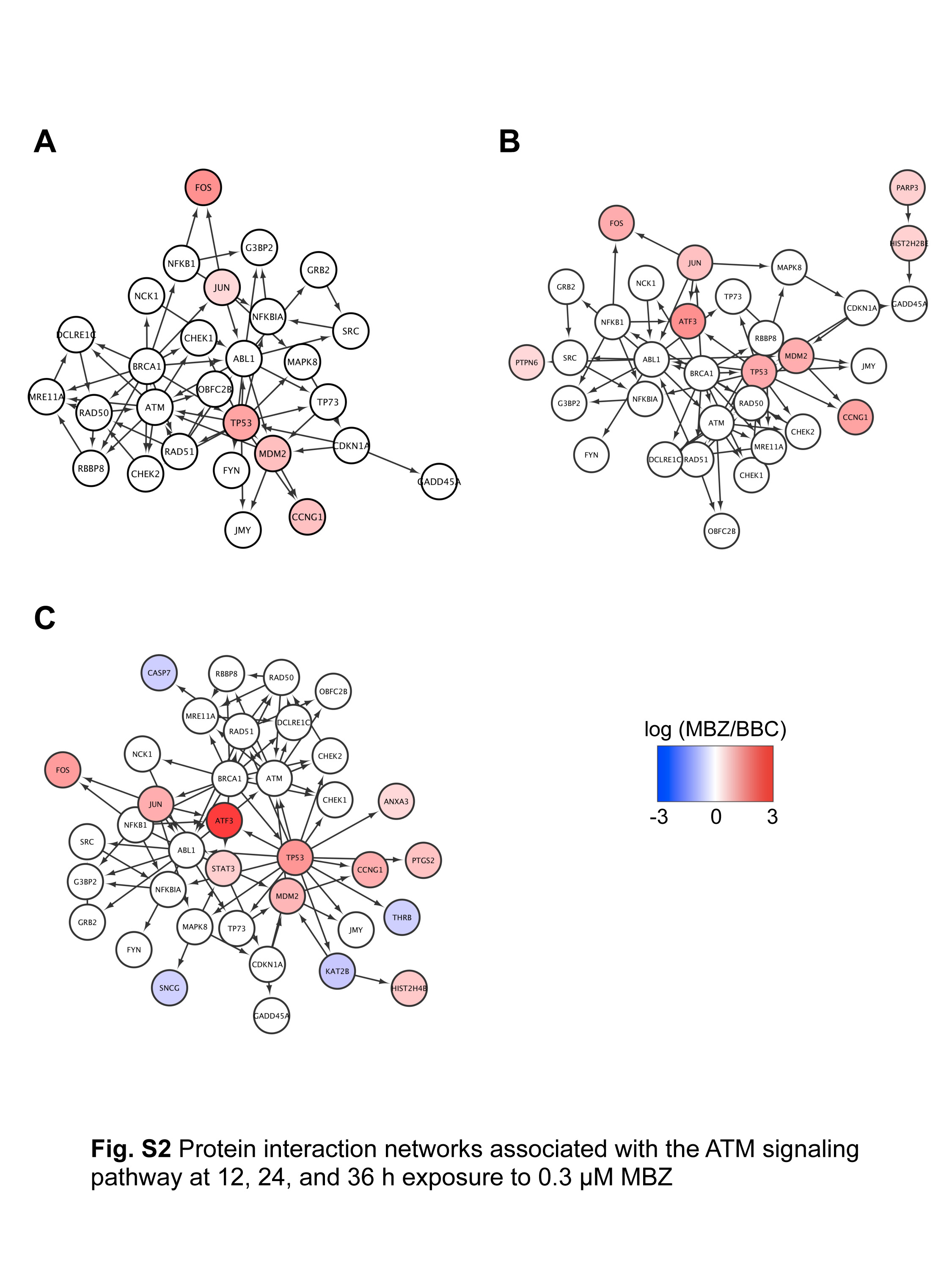

Supplement: Figure S2 — Protein interaction networks associated with the ATM signaling pathway at 12, 24, and 36 h exposure to 0.3 μM MBZ. The protein interaction networks associated with the ATM signaling pathway at 12, 24, and 36 h exposure to 0.3 μM MBZ are shown in A, B, and C, respectively. [file Image2.jpg]

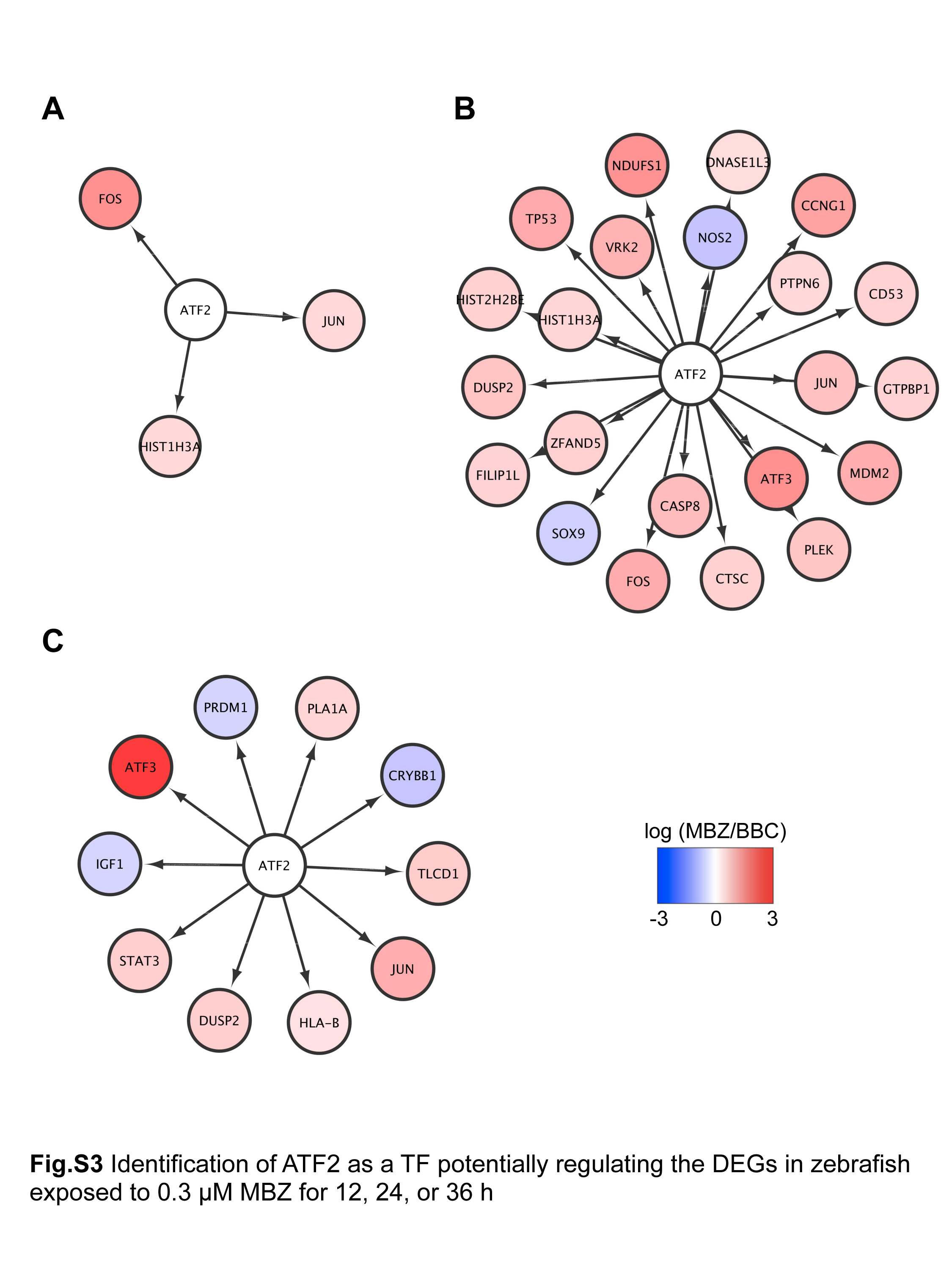

Supplement: Figure S3 — Identification of ATF2 as a TF potentially regulating the DEGs in zebrafish exposed to 0.3 μM MBZ for 12, 24, or 36 h. The networks between ATF2 and target genes at 12, 24, and 36 h exposure to 0.3 μM MBZ are shown in A, B and C, respectively. [file Image3.jpg]
